# Supplementary material for: Ice front retreat reconfigures meltwater-driven gyres modulating ocean heat delivery to an Antarctic ice shelf
Source: Nat Commun. 2022 Jan 13;13:306. doi: 10.1038/s41467-022-27968-8 (PMC8758661; doi:10.1038/s41467-022-27968-8)
Supplement: Supplementary file 1 — Supplementary Information [file 41467_2022_27968_MOESM1_ESM.pdf]

Supplementary information

Supplementary Figures

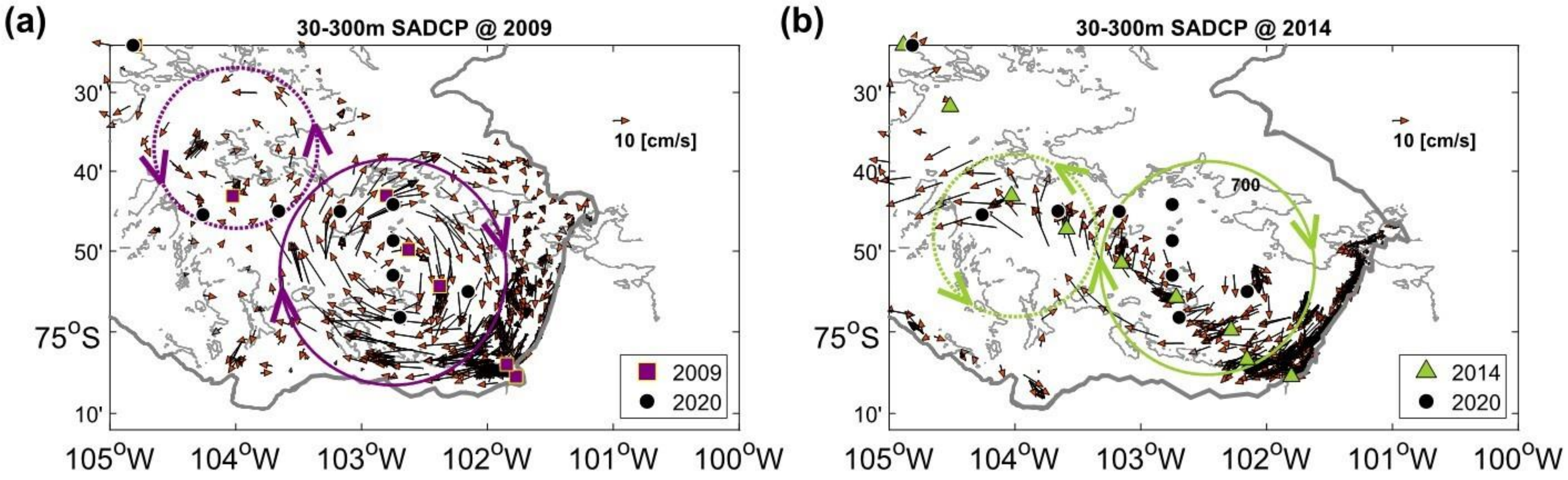

Supplementary Fig. 1. Horizontal ocean circulations in Pine Island Bay (PIB). (a) Red-faced arrows denote the ocean currents averaged over depth ranging from 30 to 300 m based on the SADCP data in 2009. The large solid purple circle indicates the approximate extent of the cyclonic gyre observed in 2009[20]. The large dotted purple circle denotes the area where the anticyclonic gyre was expected to exist in 2009. Grey contours denote 700 m isobaths. Purple squares and black circles indicate the positions of 2009 and 2020 CTD data presented in Fig. 2a, respectively. **(b)** Same as in **(a)** but for 2014. The large solid (dotted) green circle denotes the area where the cyclonic (anticyclonic) gyre was expected to exist in 2014. Green triangles and black circles indicate the positions of 2014 and 2020 CTD data presented in Fig. 2a, respectively.

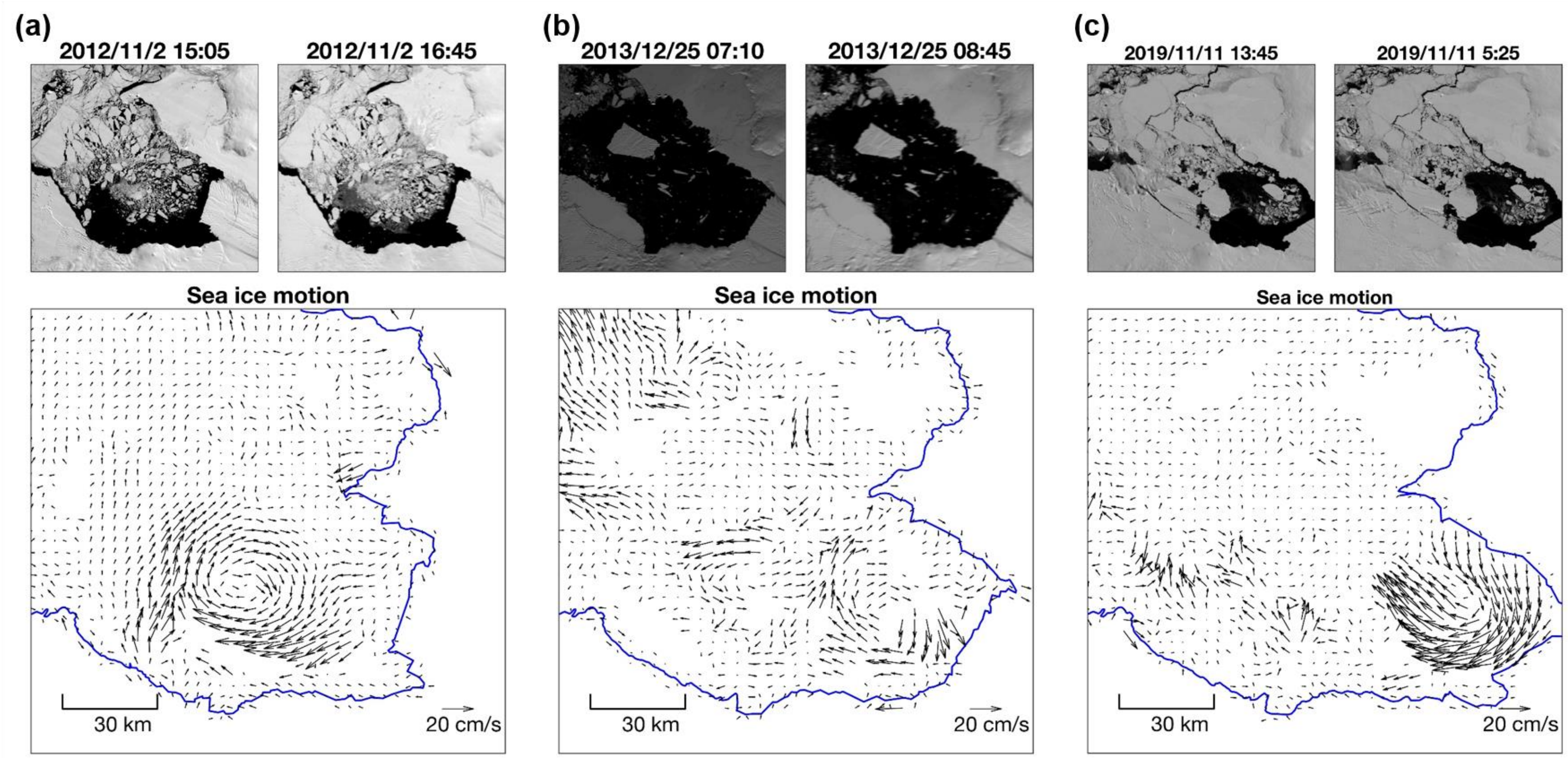

Supplementary Fig. 2. Sea ice motion derived from sea ice images (YYYY/MM/DD HH:MM). (a) Sea ice motion derived from MODIS imagery on 2 November 2012. **(b)** Same as in **(a)** but for 25 December 2013. **(c)** Same as in **(a)** but for 11 and 12 November 2019.

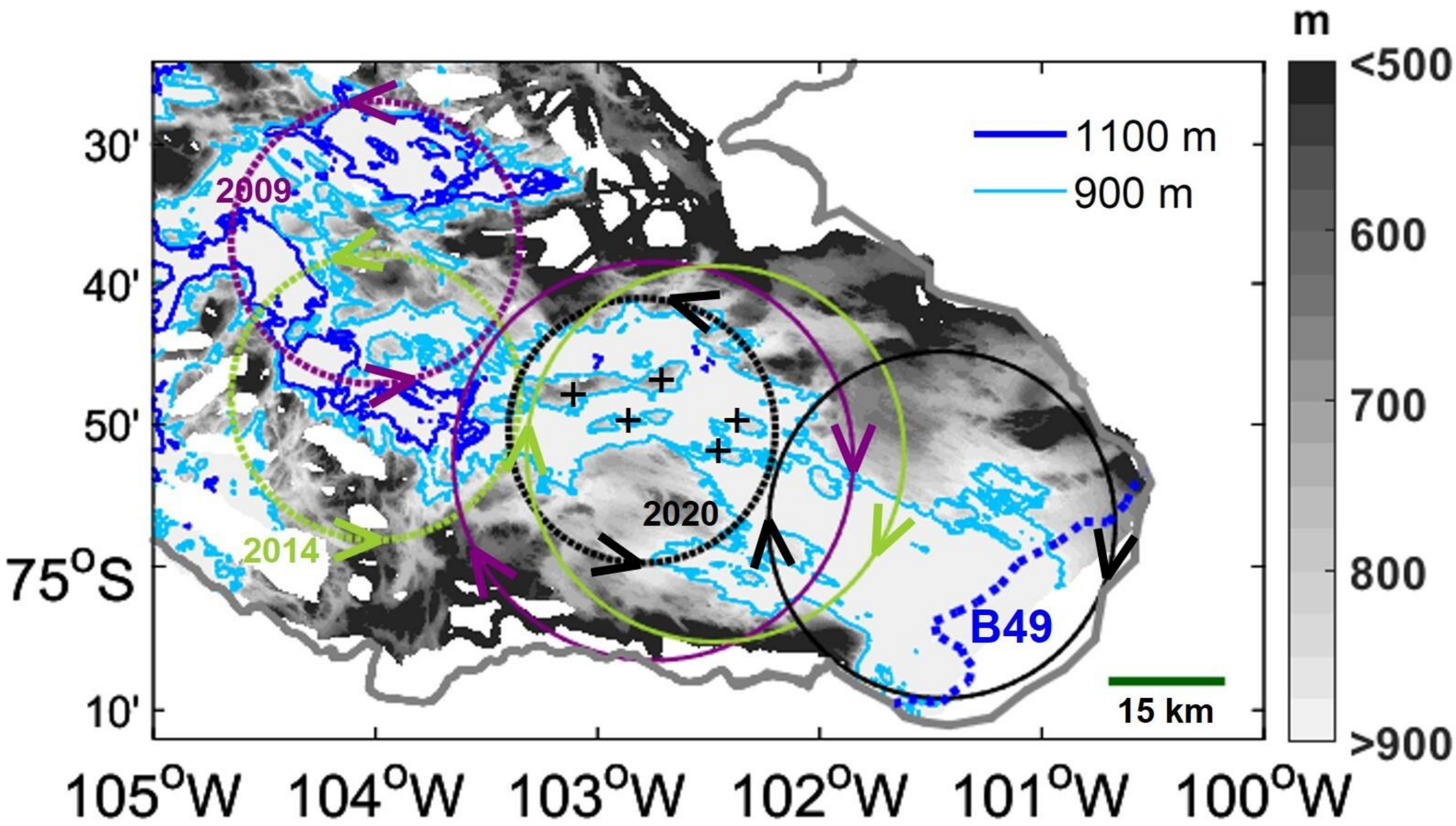

Supplementary Fig. 3. Local high-resolution bathymetric map in PIB. The large solid (dotted) purple, green, and black circles with arrows denote the approximate size and position of the cyclonic (anticyclonic) gyres in 2009, 2014, and 2020, respectively. The black cross symbols denote seabed peaks within the extent of the anticyclonic gyre in 2020. The white regions indicate that there are no high-resolution bathymetric data[22].

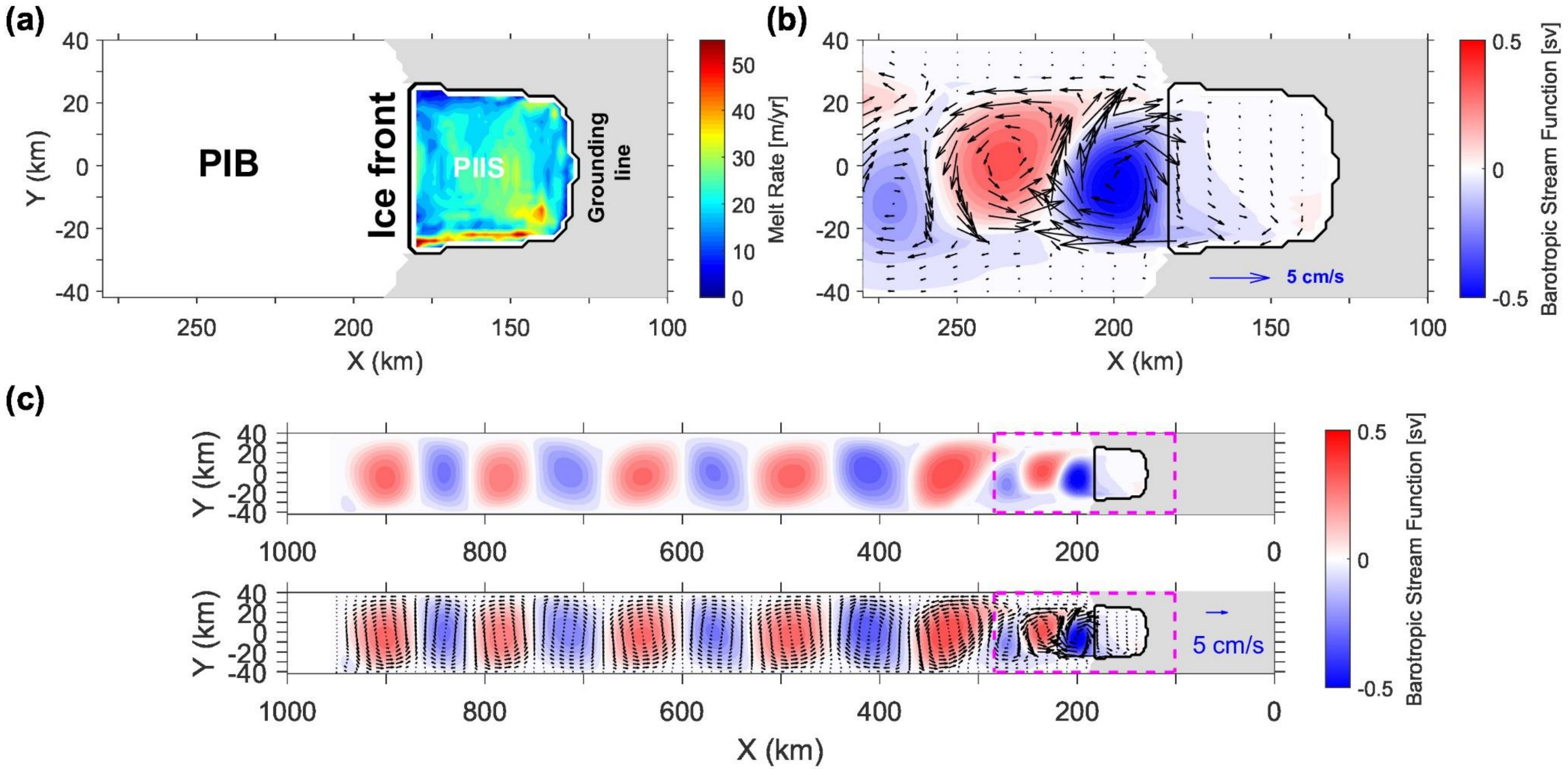

Supplementary Fig. 4. MITgcm simulation results near PIIS. (a) Distribution of averaged melt rate over the final 2 model years (70 model-year run) under PIIS and PIB. **(b)** Zoomed-in plot (the magenta box in **(c)**) of barotropic stream function and velocity fields averaged over the final 2 model years under PIIS and PIB. **(c)** Full-domain plot of barotropic stream function and velocity fields averaged over the final 2 model years under PIIS and PIB.

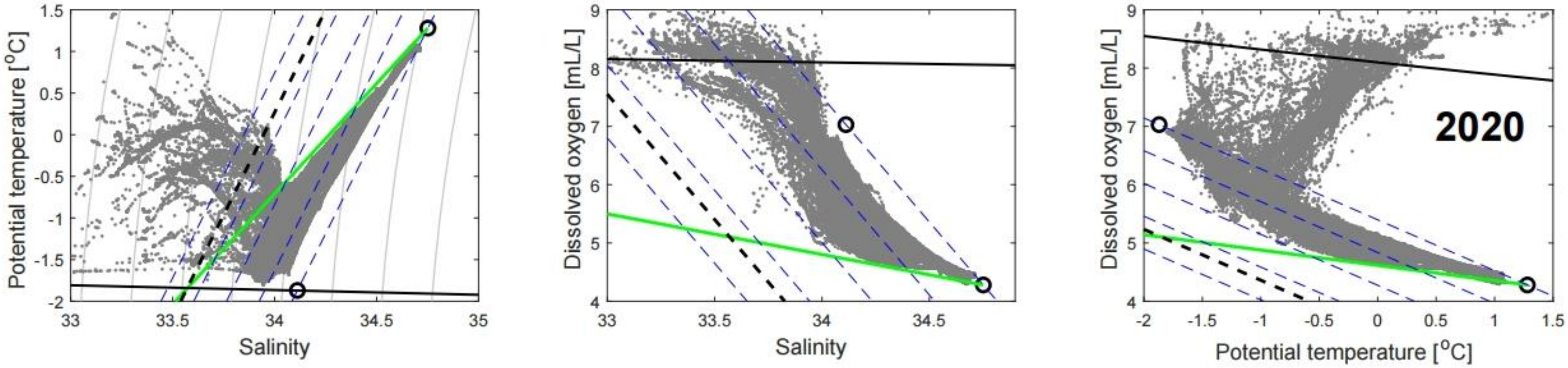

Supplementary Fig. 5. Potential temperature–salinity–dissolved oxygen (*θ*-*S*-*DO*) diagrams for 2020. The properties of winter water (WW) and modified Circumpolar Deep Water (mCDW) used for the estimation of the meltwater fraction are represented by black circles. The freezing point temperature (left-hand panel) and dissolved oxygen saturation (centre and right-hand panels) at atmospheric pressure are indicated by solid black lines, whereas the contours of the potential density (1,026.4–1,028 kg/m^3 at 0.2 kg/m^3 intervals) are denoted by solid grey lines (left-hand panel). The meltwater fraction from 0 to 40‰ at 10‰ intervals is shown by dashed blue lines. The mCDW and ice water properties are linked with green lines. The theoretical upper bounds of the meltwater fraction (intersection of green and surface freezing point lines)[14] are indicated by bold dashed black lines.

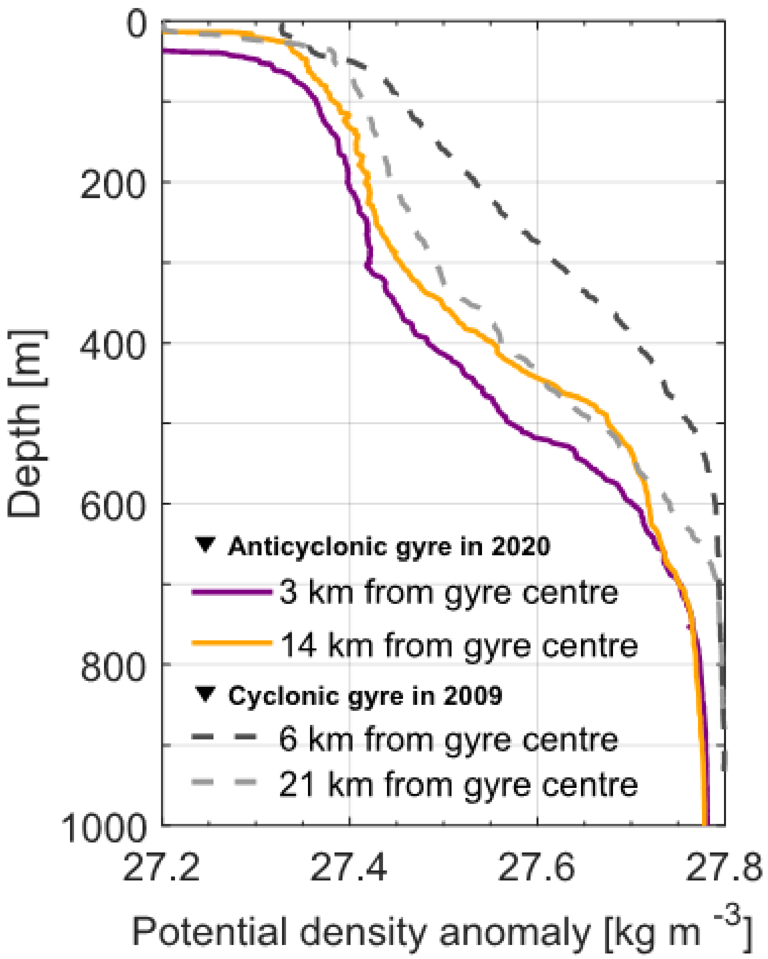

Supplementary Fig. 6. Vertical profiles of the potential density in the gyre cores. The solid (dashed) lines indicate the potential density profiles in the anticyclonic (cyclonic) gyre core in 2020 (2009).

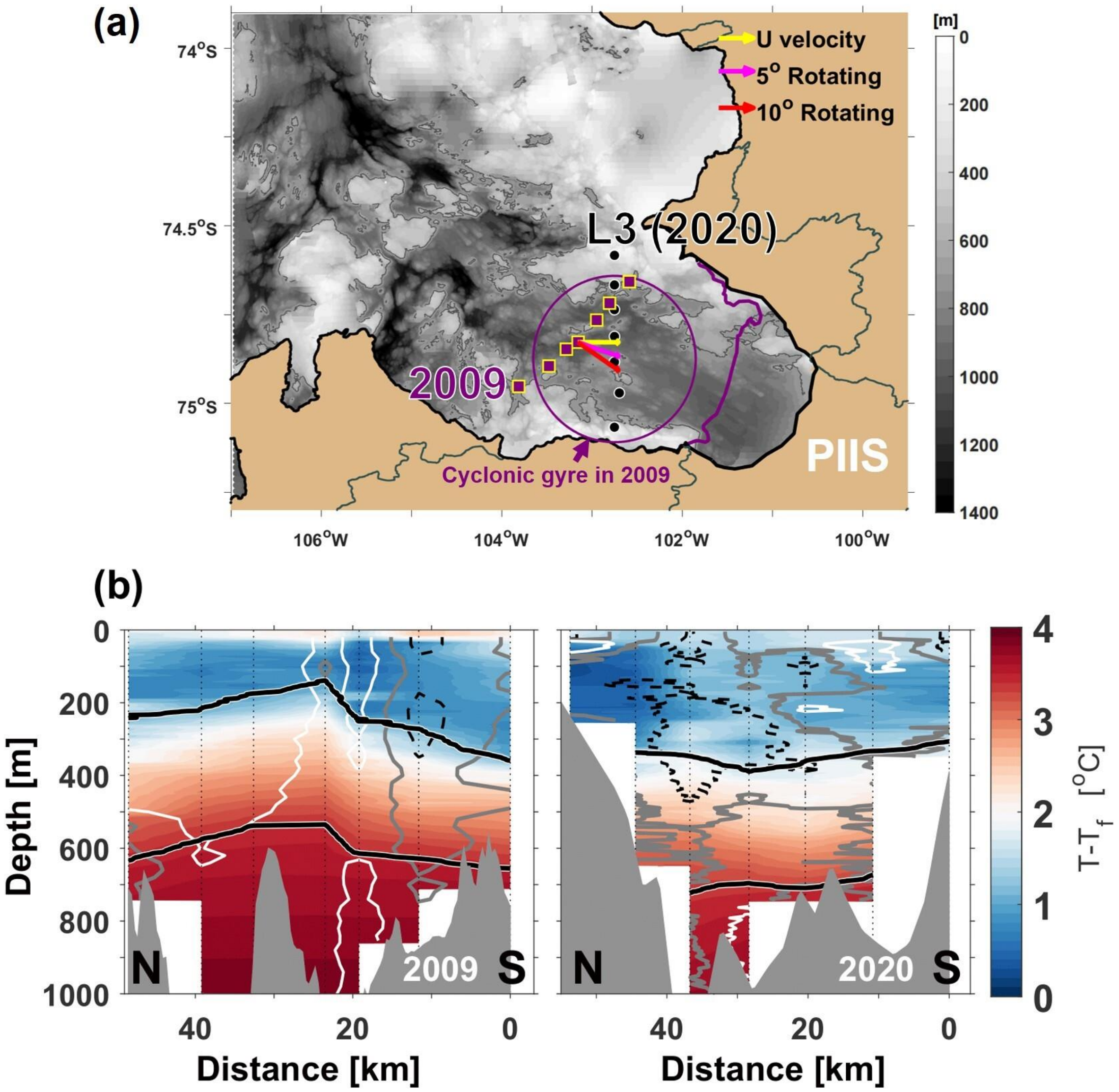

Supplementary Fig. 7. Comparison of PIB sections between 2009 and 2020. (a) Same as in Fig. 1a but for stations observed in 2009 within the extent of the cyclonic gyre. **(b)** Same as in Fig. 4b but for the CTD/LADCP sections shown in **(a)**.

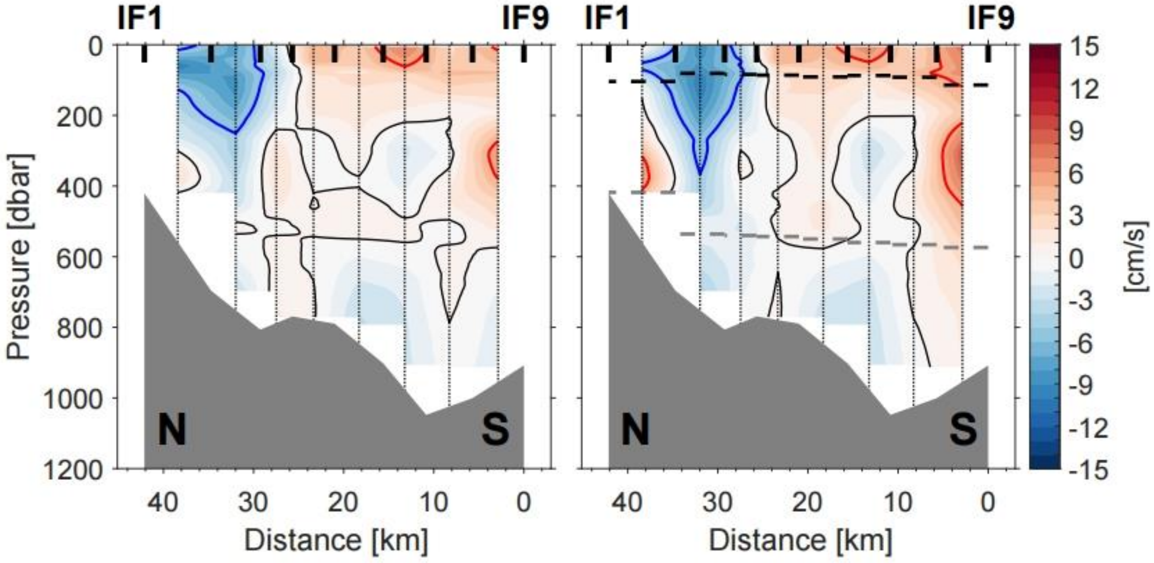

Supplementary Fig. 8. Geostrophic current velocities in front of Pine Island Ice Shelf (PIIS). The left panel indicates the geostrophic velocities perpendicular to the ice shelf front estimated using CTD data from IF1 to IF9 (Fig. 1a). Red and blue contours indicate outflow from the ice shelf and inflow to the ice shelf, respectively, at a speed of 5 cm/s. The zero velocity is indicated by a black contour. The right panel is the same as in the left panel but for geostrophic velocities adjusted using the tracer budget conservation method[8,44]. The dashed grey line indicates the initial depths of the level without motion before the adjustment. The dashed black line represents the layers that were excluded due to unreliable meltwater fractions.

Supplementary Tables

Supplementary Table 1. Mean ocean heat content in the 400–700 m and mCDW layers for each line. The value in the bracket is calculated using the data observed on 4 February 2020.

Layer | Line	L1	L2	L3	L4	IF
400–700 m	3.55 GJ	3.25 GJ	3.14 [3.11] GJ	3.40 GJ	3.41 GJ
mCDW	4.89 GJ	4.65 GJ	4.25 [4.21] GJ	3.86 GJ	3.52 GJ

Supplementary Table 2. Mean velocity [cm/s] at the mCDW layer and the number of data points used for calculation (in parentheses). The value in the bracket is calculated using the data observed on 4 February 2020. All velocity values were rounded up to the second decimal place. A positive value indicates the velocity towards the Pine Island Ice Shelf. (Upper: use velocity rotated by 5°; middle: use zonal velocity; lower: use velocity rotated by 10°)

Line | Station #	North					South		
	1	2	3	4	5	6	7	Mean
2009	–1.76<br>(94)	3.08<br>(407)	2.46<br>(508)	2.73<br>(495)	6.42<br>(456)	–0.41<br>(228)	2.55<br>(55)	2.97
L3	·<br>·	·<br>·	–1.51<br>(325)	5.32<br>(339)	0.16<br>(36)	0.04 [0.4]<br>(198 [131])	·<br>·	1.48 [1.66]
2009	–1.54	3.34	2.62	2.87	6.73	–0.50	2.68	3.15
L3	·	·	–1.29	5.49	0.13	0.15 [0.35]	·	1.65 [1.80]
2009	–1.97	2.80	2.28	2.57	6.07	–0.31	2.40	2.77
L3	·	·	–1.73	5.11	0.19	–0.07 [0.53]	·	1.30 [1.50]

Supplementary Table 3. Same as in Supplementary Table 2 but for the heat flux [TW] through the mCDW layer.

Line | Station #	North						South	
	1	2	3	4	5	6	7	Mean
2009	–0.11 (94)	1.52 (407)	1.51 (508)	1.39 (495)	2.68 (456)	–0.13 (228)	0.12 (55)	1.45
L3	· ·	· ·	–0.58 (325)	2.17 (339)	0.01 (36)	0.01 [0.08] (198 [131])	· ·	0.61 [0.67]
2009	–0.10	1.64	1.61	1.46	2.81	–0.16	0.13	1.54
L3	·	·	–0.49	2.23	0.01	0.05 [0.07]	·	0.68 [0.73]
2009	–0.12	1.38	1.40	1.31	2.53	–0.10	0.11	1.36
L3	·	·	–0.66	2.08	0.01	–0.02 [0.10]	·	0.54 [0.61]

Supplementary Table 4. Ocean heat content [GJ] at 400–700 m and number of data points used for calculation (in parentheses) at each station. All ocean heat content values were rounded to the second decimal place. The value in the bracket is calculated using the data observed on 4 February 2020.

Line |	North								South
Station #	1	2	3	4	5	6	7	8	9
L1	3.69	3.70	3.60	3.55	3.47	3.26	·	·	
	(301)	(301)	(301)	(301)	(301)	(301)	·	·	
L2	·	·	3.45	3.22	3.22	3.09	·		
	·	·	(301)	(301)	(301)	(289)	·		
L3	·	2.67	3.40	3.21	3.06	3.29 [3.12]	·		
	·	(249)	(301)	(301)	(301)	(301 [301])	·		
L4	2.64	3.62	3.66	3.58	3.52	3.37	3.25		
	(232)	(301)	(301)	(301)	(301)	(301)	(301)		
IF	·	3.37	3.46	3.50	3.50	3.36	3.35	3.42	3.31
	·	(289)	(301)	(301)	(301)	(301)	(301)	(301)	(301)

Supplementary Table 5. Same as in Supplementary Table 4 but for the mCDW layer.

Line |	North								South
Station #	1	2	3	4	5	6	7	8	9
L1	2.90	0.28	3.84	6.05	6.49	2.00	·	·	
	(205)	(20)	(268)	(413)	(440)	(140)	·	·	
L2	·	·	4.73	5.21	3.78	·	·		
	·	·	(323)	(354)	(260)	·	·		
L3	·	·	4.77	4.97	0.50	2.84 [1.87]	·		
	·	·	(325)	(339)	(36)	(198 [131])	·		
L4	·	2.25	5.22	3.60	4.19	3.17	0.50		
	·	(161)	(364)	(251)	(290)	(219)	(36)		
IF	·	1.25	2.19	1.83	2.26	3.38	5.33	4.60	2.87
	·	(90)	(156)	(131)	(161)	(236)	(364)	(317)	(200)
